# New metabolic pathway for degradation of 2-nitrobenzoate by *Arthrobacter* sp. SPG

**DOI:** 10.3389/fmicb.2015.00551

**Published:** 2015-06-02

**Authors:** Pankaj K. Arora, Ashutosh Sharma

**Affiliations:** ^1^School of Biotechnology, Yeungnam UniversityGyeongsan, South Korea; ^2^Escuela de Ingenieria en Alimentos, Biotecnologia y Agronomia, Instituto Tecnológico y de Estudios Superiores de MonterreySan Pablo, Mexico

**Keywords:** 2-nitrobenzoate, 3-nitrobenzoate, 4-nitrobenzoate, salicylate, catechol

## Abstract

*Arthrobacter* sp. SPG utilized 2-nitrobenzoate as its sole source of carbon and energy and degraded it with accumulation of stoichiometric amounts of nitrite ions. Salicylate and catechol were detected as metabolites of the 2-nitrobenzoate degradation using high performance liquid chromatography and gas chromatography–mass spectrometry. Enzyme activities for 2-nitrobenzoate-2-monooxygenase, salicylate hydroxylase, and catechol-1,2-dioxygenase were detected in the crude extracts of the 2-nitrobenzoate-induced cells of strain SPG. The 2-nitrobenzoate-monooxygenase activity resulted in formation of salicylate and nitrite from 2-nitrobenzoate, whereas salicylate hydroxylase catalyzed the conversion of salicylate to catechol. The ring-cleaving enzyme, catechol-1,2-dioxygenase cleaved catechol to *cis,cis*-muconic acid. Cells of strain SPG were able to degrade 2-nitrobenzoate in sterile as well as non-sterile soil microcosms. The results of microcosm studies showed that strain SPG degraded more than 90% of 2-nitrobenzoate within 10–12 days. This study clearly shows that *Arthrobacter* sp. SPG degraded 2-nitrobenzoate via a new pathway with formation of salicylate and catechol as metabolites. *Arthrobacter* sp. SPG may be used for bioremediation of 2-nitrobenzoate-contaminated sites due to its ability to degrade 2-nitrobenzoate in soil.

## Introduction

Nitrobenzoates (NBs) including 2-nitrobenzoate (2NB), 3-nitrobenzoate (3NB), and 4-nitrobenzoate (4NB) are used in the synthesis of chemicals, pharmaceuticals and dyes (Zylstra et al., 2000). NBs are white to light yellow colored crystals, which are insoluble in water, but soluble in alcohol. These compounds are toxic to living beings because of their genotoxicity, mutagenicity, and hematologic toxicity (Sundvall et al., 1984; NTP, 1994; Grummt et al., 2006).

Bacterial degradation of NBs proceeds either via the oxidative route or through the reductive route (Nadeau and Spain, 1995; Chauhan and Jain, 2000; Hasegawa et al., 2000; Pandey et al., 2003). The oxidative pathway involves an oxygenase-dependent removal of the nitro group as nitrite (Nadeau and Spain, 1995), whereas the initial step in the reductive pathway is a nitroreductase-catalyzed reaction resulting in ammonium release (Chauhan and Jain, 2000; Hasegawa et al., 2000; Pandey et al., 2003).

Thus far, bacterial 2NB and 4NB degradations have been found to proceed exclusively via the reductive pathway (Groenewegen et al., 1992; Haigler and Spain, 1993; Rhys-Williams et al., 1993; Yabannavar and Zylstra, 1995; Chauhan and Jain, 2000; Hasegawa et al., 2000; James et al., 2000; Samanta et al., 2000; Pandey et al., 2003). The reductive pathway of the 4NB degradation has been studied in a number of bacteria including *Comamonas acidovorans* NBA-10 (Groenewegen et al., 1992), *Ralstonia pickettii* YH105 (Yabannavar and Zylstra, 1995), *Ralstonia* sp. SJ98 (Samanta et al., 2000), *Pseudomonas* sp. strain 4NT (Haigler and Spain, 1993), and *Pseudomonas putida* TW3 (Rhys-Williams et al., 1993; James et al., 2000). The initial step of the reductive pathway involves conversion of 4NB to 4-hydroxylaminobenzoate by a 4NB-reductase. In next step, 4-hydroxylaminobenzoate lyase catalyzes deamination of 4-hydroxylaminobenzoate with the concomitant formation of protocatechuate. The genes involved in this pathway have also been cloned and characterized in *Pseudomonas putida* TW3 and *Pseudomonas pickettii* YH105 (Yabannavar and Zylstra, 1995; Hughes and Williams, 2001).

The reductive pathway of the 2NB degradation proceeds either via 3-hydroxyanthranilate or via anthranilate (Chauhan and Jain, 2000; Hasegawa et al., 2000; Pandey et al., 2003). The 3-hydroxyanthranilate pathway is well-studied in *Pseudomonas fluorescens* KU-7 in which 2NB degradation is initiated with the reduction of 2NB to 2-hydroxylaminobenzoate by a NADH:FMN reductase (Hasegawa et al., 2000). The next step, catalyzed by a mutase, involves conversion of 2-hydroxylaminobenzoate to 3-hydroxyanthranilate that undergoes ring cleavage by a 3-hydroxyanthranilate-3,4-dioxygenase to produce 2-amino-3-carboxymuconate-6-semialdehyde. In the next step, a decar boxylase catalyzes decarboxylation of 2-amino-3-carboxy muconate-6-semialdehyde to 2-aminomuconic semialdehyde, which undergoes further degradation to produce pyruvate and acetaldehyde (Hasegawa et al., 2000).

The anthranilate pathway of 2NB degradation was observed in *Burkholderia* sp. SJ98 (previously known as *Ralstonia* sp. strain SJ98) that degrades it via o-hydroxylaminobenzoate and anthranilate (Samanta et al., 2000). Another bacterium, *Arthrobacter protophormiae* RKJ100 degrades 2NB via both 3-hydroxyanthranilate and anthranilate pathways (Pandey et al., 2003). Although, several studies described the bacterial degradation pathways of 2NB, there is no report on oxidative degradation pathway of 2NB. In this study, oxidative pathway of 2NB degradation has been reported via salicylate and catechol by a previously isolated bacterium, *Arthrobacter* sp. SPG.

In past, several bacteria have been reported for their ability to degrade various nitroaromatics in soil, which is prerequisite for bio-augmentation of contaminated soil (Arora et al., 2014a). Despite the fact that NBs are wide spread in soil, there is no single strain of NBs-degrading bacterium that could degrade NBs in soil. *Arthrobacter* sp. SPG has also been reported for its ability to degrade nitroaromatics in soil (Arora et al., 2014b); therefore, the potential of *Arthrobacter* sp. SPG to degrade 2NB in soil has also investigated using soil microcosms in this study.

## Materials and Methods

### Bacterial Growth and 2NB Degradation

A bacterium, *Arthrobacter* sp. SPG used in this study was previously isolated from a pesticide contaminated site of India and able to utilize 2NB as its sole source of carbon and energy (Arora, 2012). Strain SPG was grown on 250 ml Erlenmeyer flasks on 100 ml minimal medium supplemented with 0.5 mM 2NB. The minimal media was composed of Na_2_HPO_4_ (4 g/L), KH_2_PO_4_ (2 g/L), (NH_4_)_2_SO_4_ (0.8 g/L), MgSO_4_.7H_2_O (0.8 g/L), and trace element solution. The trace element solution was prepared freshly as described previously (Arora and Jain, 2012). The pH of minimal medium was adjusted at 7.0 pH using 5 M NaOH and medium was autoclaved at 15 psi for 20 min. The autoclaved minimal medium was allowed to cool at room temperature and the filter sterilized 2NB was added into the medium at the concentration of 0.5 mM. The stock solution of 2NB was prepared in methanol at the concentration of 100 mM. The incubation was carried out under shaking conditions (200 rpm) at ambient temperature. Bacterial cultures (samples, 3 ml) were collected at every 4 h up to the 48 h for analysis of bacterial growth, 2NB depletion, nitrite release and ammonia release. The bacterial growth was measured in spectrophotometer by an increase in optical density (OD) at 600 nm. To monitor 2NB depletion and detection of the nitrite and ammonia ions, samples (2 ml) collected at regular intervals were centrifuged at 10000 rpm for 5 min and supernatants were used for further analysis. 2NB depletion was monitored with decrease in absorbance at 268 nm using UV-visible spectrophotometer (Perkin Elmer; Chauhan and Jain, 2000). The residual amounts was calculated from a standard curve prepared using authentic 2NB. The nitrite and ammonia releases were monitored by the methods as described previously (Arora and Jain, 2012).

### Identification of Metabolite(s)

To identify the metabolite of the degradation of 2NB, strain SPG was grown on 1 L Erlenmeyer flask containing 500 ml minimal media and 0.5 mM 2NB as its sole source of carbon energy. Samples (50 ml) were collected at regular intervals (0, 12, 24, and 36 h) and centrifuged at 10000 rpm for 15 min. The supernatants were extracted with ethyl acetate as described previously (Arora and Jain, 2012). The extracted samples were dissolved in 20 μl methanol, and subjected to high performance liquid chromatography (HPLC) and gas chromatography–mass spectrometry (GC–MS) for analysis.

A Waters 600 model HPLC system was used for this study. This system was equipped with a photodiode array detector system. Separation was performed on C18 reverse-phase silica column with a solvent [methanol: water (20:80, v/v) containing 1% glacial acetic acid] at flow rate of 1.0 ml/min.

GC–MS was carried out using Agilent Gas Chromatography system equipped with Time-of-Flight Mass Spectrometer and HP-5 column with ionization of 70 eV (Arora et al., 2014c). The column temperature was initially increased from 50 to 150^°^C at the rate of 5^°^C/min and then from 150 to 280^°^C at the rate of 10^°^C/min. The carrier gas (helium) flow rate was 1.5 ml/min. The temperature of the transfer line was 225^°^C, whereas temperature of ion source was 250^°^C (Arora et al., 2014c).

### Enzyme Assay for 2-Nitrobenzoate-2-Monooxygenase

The enzyme, 2NB-2-monooxygenase catalyzed conversion of 2NB to salicylate with release of nitrite ions. The enzyme activity was determined by measuring nitrite ions released from 2NB upon incubation with cell-free lysate containing 50 mM Tris-HCl buffer (pH 8.0), 0.2 mM NADH (nicotinamide adenine dinucleotide, reduced form) 0.08 mM FAD (flavin adenine dinucleotide), 1 mM MgSO_4_, 20 mg of cell-free lysate, and 500 μM 2NB in a total reaction volume of 5 ml. The reaction was initiated by the addition of 2NB in the reaction mixture and the sample (500 μl) was collected after 15 min of incubation and subjected to quantitation of the nitrite release in reaction mixture. The remaining sample was extracted with ethyl acetate and analyzed by GC–MS for identification of final product. The cell free lysate was prepared as described previously using 2NB (Arora and Jain, 2012).

### Enzyme Assay for Salicylate Hydroxylase

Salicylate hydroxylase catalyzes the conversion of salicylate to catechol. The salicylate hydroxylse activity was performed with UV-visible spectrophotometer (Perkin Elmer) and reaction mixture containing 50 mM Tris-HCl (pH 8.0) buffer, 1 mM EDTA, 200 μM NADH, 150 μM salicylate, and 50 μl of cell extracts in a volume of 3 ml. The NADPH-oxidation was monitored in decrease in the absorbance at 340 nm. The conversion of salicylate to catechol was monitored spectrophotometrically as described by White-Stevens and Kamin (1972). The decrease of the absorbance of salicylate was monitored at 296 nm, whereas the catechol formation was monitored at 276 nm.

### Enzyme Assay for Catechol-1,2-Dioxygenase

Catechol-1,2-dioxygenase activity was determined spectrophotometrically by measuring the increase of absorbance at 260 nm due to the cleavage of catechol into *cis,cis*-muconate. The reaction mixture contained 50 μmol of 50 mM Tris-HCl (pH 7.4) buffer, and 200 μM of catechol in a total volume of 1 ml. After addition of crude extracts, the increase at 260 nm (corresponding to the formation of *cis,cis*-muconate) was measured in a silica cuvette with a 1.0-cm light path at spectrophotometer.

### Microcosm Studies

Microcosm studies were carried out to monitor the 2NB degradation in soil by *Arthrobacter* sp. SPG. Soil (pH 7.2, 2.4% organic matter content and 30% moisture content) was collected from the University campus. Microcosms were prepared in 100 ml beakers containing 20 g soil artificially contaminated with 100 ppm 2NB. Four sets of experiments were performed in this study: (a) sterile soil microcosm inoculated with strain SPG at ∼2 × 10^8^ cells colony-forming units (CFUs) g^-1^ soil; (b) non-sterile soil microcosm inoculated with strain SPG at ∼2 × 10^8^ cells CFUs g^-1^ soil; (c) control sterile microcosm without bacterial inoculation; and (d) Control non-sterile microcosm without bacterial inoculation. All beakers covered with aluminum foil were maintained under sterile conditions at 30^°^C for 10 days. Distilled water (5–10 ml) was added at regular interval to maintain the water contents in soil. Soil samples (1 g) were removed at regular intervals up to 12 days and extracted as described previously (Arora and Jain, 2012). The extracted samples were analyzed by HPLC as described above. All experiments were carried out in triplicate and under optimum conditions, which include inoculum size (2 × 10^8^ CFU g^-1^ soil), pH (7.0), temperature (30^°^C), and 2NB concentration (100 ppm).

## Results

### Bacterial Growth and 2NB Degradation

**FIGURE 1:**
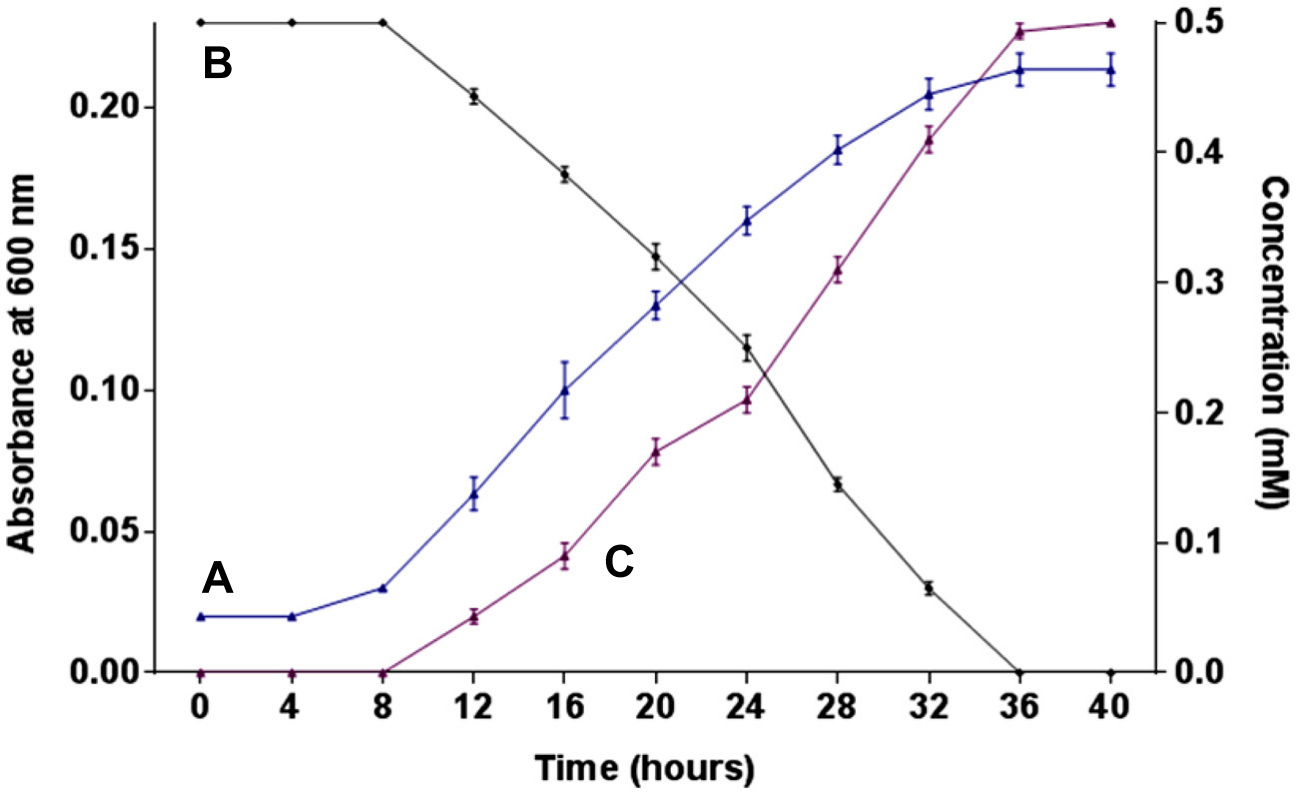
**Growth, degradation, and nitrite release. (A)** Growth of strain SPG in minimal medium containing 0.5 mM 2NB; **(B)** 2NB degradation by strain SPG; and **(C)** release of nitrite ions during bacterial degradation of 2NB.

The growth of strain SPG was monitored in minimal medium containing 0.5 mM 2NB as its sole source of carbon and energy. Bacterial growth was directly correlated with 2NB degradation by *Arthrobacter* sp. SPG. Neither bacterial growth nor 2NB degradation was observed at initial 8 h when the bacterial cells were in lag phase of growth cycle (**Figures 1A,B**). Cells of strain SPG grew after 8 h of the incubation and degraded almost 75% 2NB in 28 h of incubation. The complete degradation of 2NB has achieved after the incubation of 36 h when the bacterial cells reached at stationary phase. The stoichiometric amounts of nitrite ions (0.5 mM) were detected during the degradation of 2NB by *Arthrobacter* sp. SPG (**Figures 1C**).

### Identification of Metabolites

**FIGURE 2:**
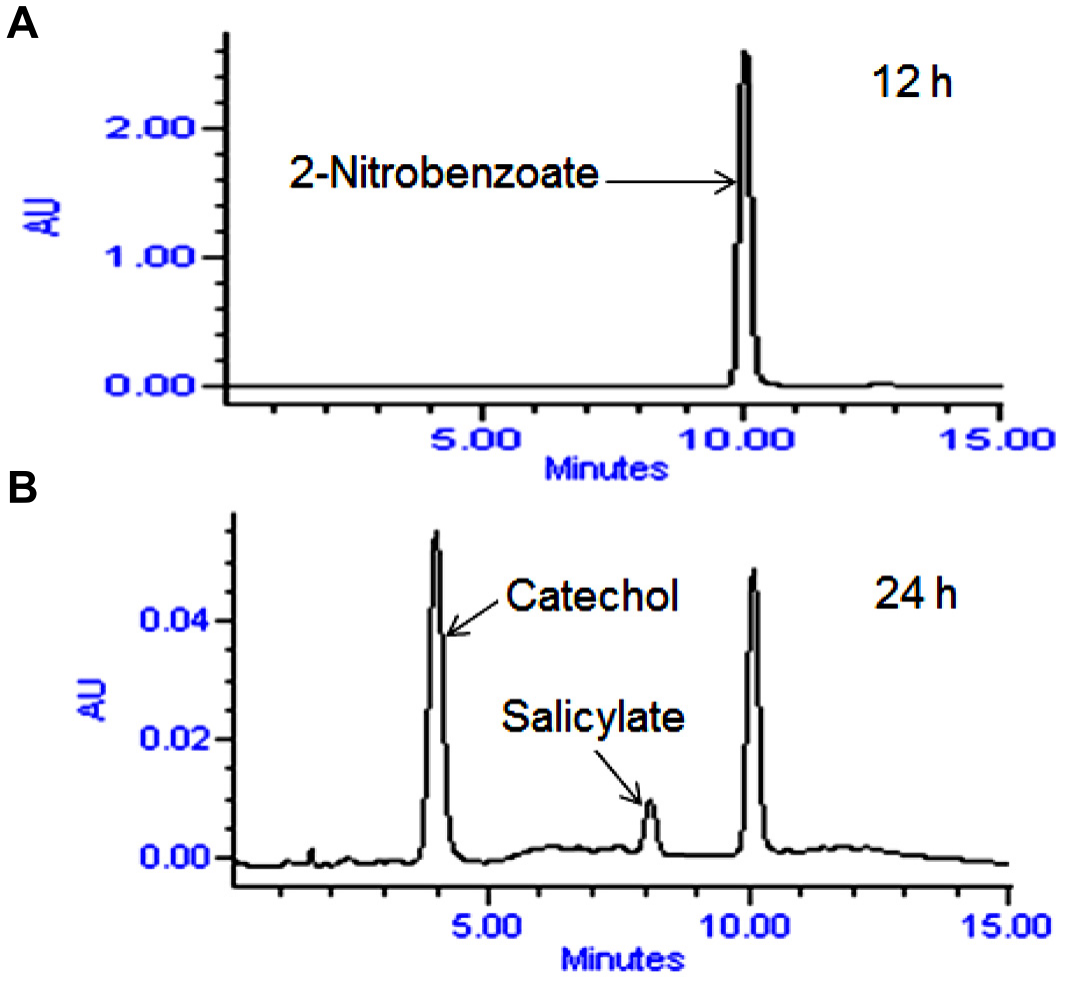
**High performance liquid chromatography (HPLC) elution profiles of the samples during the 2NB degradation. (A)** 12 h sample showing only the peak of 2NB; and **(B)** 24 h sample showing the peaks of two metabolites along with the 2NB.

**FIGURE 3:**
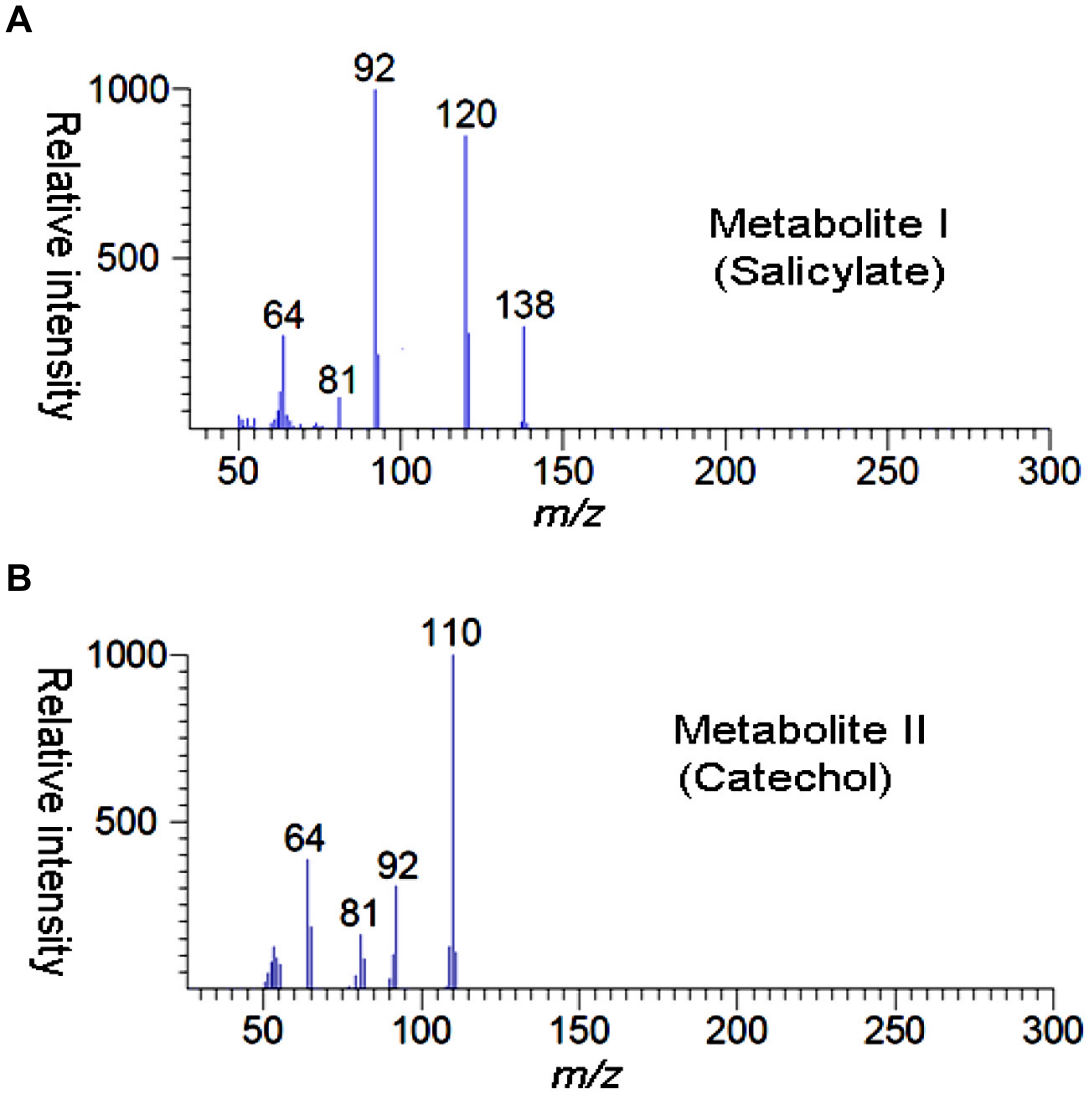
**GC–MS spectra of metabolite I **(A)** and metabolite II (B)**.

High performance liquid chromatography studies confirmed the complete degradation of the 2NB within 36 h. In the sample of 12 h, only peak of 2NB was detected (**Figures 2A**), while peaks of two metabolites in addition to 2NB peak were detected in sample collected at 24 h (**Figures 2B**). The retention time of the metabolite I and II corresponded to those of authentic standards of the salicylate and catechol. In the sample of 36 h, neither peak of the 2NB or any metabolite was detected suggesting the complete utilization of 2NB. To identify the metabolites, GC–MS analysis was carried out. The mass spectra of metabolite I had a molecular ion peak at *m/z* 138 and other major fragments were observed at *m/z* 120 and 119 (**Figures 3A**). The mass spectra of metabolite II contained a main peak at *m/z* 110 and other fragments were observed at *m/z* 92, 81, and 64 (**Figures 3B**). On the basis of the NIST (National Institute of Standards and Technology) library match, metabolites I and II were identified as salicylate and catechol, respectively.

### Enzyme Assays

In the crude extracts of the 2NB-induced cells of strain SPG, we have detected enzyme activities for 2NB-2-monooxygenase, salicylate hydroxylase, and catechol-1,2-dioxygenase. The activity of 2NB-2-monoxygenase was confirmed by detection of salicylate using GC–MS and release of nitrite ions in reaction mixture. The salicylate hydroxylase activity was confirmed by the detection of catechol using a spectrophotometer. The catechol-1, 2-dioxygenase activity was confirmed spectrophotometrically with the increase in absorbance at 260 nm (corresponding to formation of *cis,cis*-muconate).

### Microcosm Studies

**FIGURE 4:**
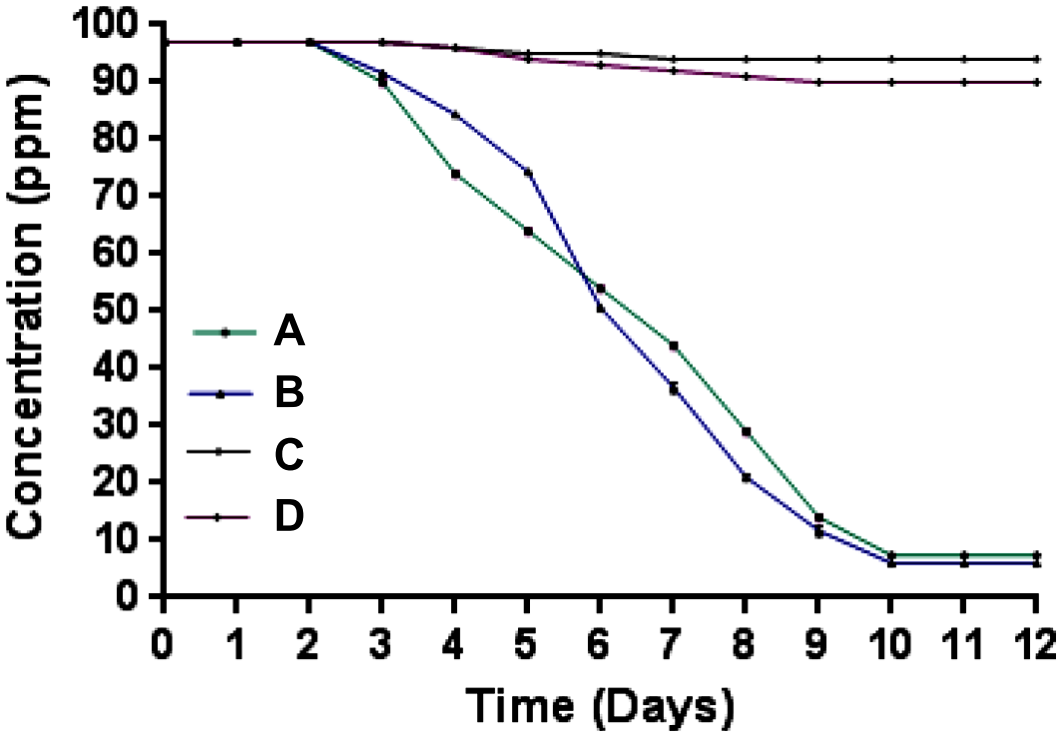
**Microcosm studies. (A)** Sterile soil microcosm with inoculated bacteria; **(B)** non-sterile soil microcosm with inoculated bacteria; **(C)** control sterile microcosm without bacterial inoculation, and **(D)** control non-sterile microcosm without bacterial inoculation.

Microcosm studies showed that cells of strain SPG degraded more than 90% of 2NB in sterile and non-sterile soli within 10–12 days (**Figure 4**). There was no 2NB degradation at initial 2 days in sterile as well as non-sterile soil microcosms. In sterile microcosm, the degradation was 9 and 25% at 3 and 4 days, respectively. After 6 days, the degradation rate was 45%. Almost 70% degradation was observed after 8 days. At 10 days, more than 90% NB was degraded by cells of strain SPG. In non-sterile soil, the 2NB degradation was 47, 65, and 80% in 6, 7, and 8 days, respectively. More than 90% degradation was observed after 10 days. Only 5–10% degradation was observed in both control microcosms within 12 days.

## Discussion

**FIGURE 5:**
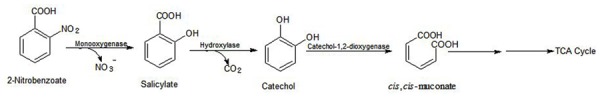
**Proposed pathway of the 2NB degradation for *Arthrobacter* sp. SPG**.

*Arthrobacter* sp. SPG utilizes 2NB as its sole source of carbon and energy and degrades it with stoichiometric release of nitrite ions. Initially, a 2NB-monooxygenase catalyzes conversion of 2NB to salicylate with release of nitrite ions. The next step, catalyzed by salicylate hydroxylase, involves conversion of salicylate to catechol, which undergoes *ortho* ring cleavage by a catechol-1,2-dioxygenase to produce *cis,cis*-muconic acid (**Figure 5**).

Previous studies show that bacterial degradation of NBs (4NB, 2NB, and 3NB) occurs either via reductive pathway with accumulation of ammonium ions (Groenewegen et al., 1992; Haigler and Spain, 1993; Rhys-Williams et al., 1993; Yabannavar and Zylstra, 1995; Chauhan and Jain, 2000; Hasegawa et al., 2000; James et al., 2000; Samanta et al., 2000; Pandey et al., 2003) or via oxidative pathway with release of nitrite ions (Nadeau and Spain, 1995). The 4NB degradation is well-characterized in *Pseudomonas putida* TW3 that degrades 4NB reductively with formation of 4-hydroxylaminobenzoate and protocatechuate (Rhys-Williams et al., 1993; James et al., 2000). The reductive degradation of 2NB proceeds via the 3-hydroxyanthranilate pathway or the anthranilate pathway (Chauhan and Jain, 2000; Hasegawa et al., 2000; Pandey et al., 2003). In this study, neither 3-hydroxyanathranilate nor anthranilate was detected as metabolite of the 2NB degradation in strain SPG. Furthermore, the reductive degradation of 2NB and 4NB proceeds with accumulation of ammonium ions (Groenewegen et al., 1992; Haigler and Spain, 1993; Rhys-Williams et al., 1993; Yabannavar and Zylstra, 1995; Chauhan and Jain, 2000; Hasegawa et al., 2000; James et al., 2000; Samanta et al., 2000; Pandey et al., 2003), whereas the 2NB degradation proceeds via release of nitrite ions in this SPG, suggesting the involvement of oxidative pathway. The 2NB-2-monooxygenase activity was observed in the crude extracts of the 2NB-induced cells of strain SPG that confirms oxidative release of nitrite ions from 2NB. These results indicate that 2NB degradation occurs via oxidative pathway in strain SPG. Nadeau and Spain (1995) reported a dioxygenase catalyzed oxidation of 3NB to protocatechuate with stoichiometric release of nitrite in *Pseudomonas* sp. strain JS51. In this study, a monooxygenase catalyzes oxidation of 2NB with stoichiometric release of nitrite in strain SPG.

Salicylate was detected as a metabolite in 2NB degradation by stain SPG. Previously, salicylate as a metabolite in the degradation pathway of several aromatic compounds, including 4-chloro-2-nitrobenzoate (Prakash et al., 2010), naphthalene (Pumphrey and Madsen, 2007), phenanthrene (Ghosal et al., 2010) and 4-chloroindole (Arora and Bae, 2015), was detected. The bacterial salicylate degradation occurs via one of the following mechanisms: (i) with formation of catechol (Pumphrey and Madsen, 2007); (ii) with formation of gentisate (Pumphrey and Madsen, 2007); and (iii) ring cleavage of salicylate (Hintner et al., 2001). In this study, catechol was detected as a metabolite, suggesting the catechol pathway should be involved in the 2NB degradation pathway. Furthermore, the salicylate hydroxylase activity was detected in the crude extracts of the 2NB induced cells of strain SPG that confirmed the formation of catechol from salicylate in the degradation pathway of the 2NB. The oxidation of salicylate to catechol has also been observed in a yeast *Trichosporon cutaneum* (Sze and Dagley, 1984). Iwasaki et al. (2010) reported a new pathway of the salicylate degradation in the yeast *Trichosporon moniliiforme* WU-0401 that degraded salicylate via phenol as the key intermediate. However, phenol was not detected as a metabolite in the 2NB degradation pathway of strain SPG.

In this study, catechol was detected as a ring cleavage substrate in the degradation pathway of 2NB. Literature studies showed that catechol is a common intermediate in the degradation pathway of several aromatic compounds (Dagley and Gibson, 1965; Gibson et al., 1968; Zeyaullah et al., 2009). The catechol degradation occurs via either *ortho*-ring cleavage by catechol-1,2-dioxygenase or *meta*-ring cleavage by catechol-2,3-dioxygenase (Zeyaullah et al., 2009). In this study, the activity of catechol-1, 2-dioxygenase was observed, suggesting the involvement of the *ortho*-cleavage pathway of catechol in the degradation of 2NB.

The 2NB degradation pathway studied in strain SPG differs from all previously reported bacterial degradation pathways of the 2NB. Strain SPG degrades 2NB via oxidatively with release of nitrite ions whereas previous studies show that 2NB bacterial degradation occurs reductively via accumulation of ammonium ions (Chauhan and Jain, 2000; Hasegawa et al., 2000; Pandey et al., 2003). The comparison of the 2NB degradation pathway of strain SPG with the degradation pathway of 4-chloro-2-nitrobenzoate in *Acinetobacter* sp. RKJ12 show that the degradation of the both of the compounds proceeds via the formation of salicylate and catechol (Prakash et al., 2010). However, the mechanism of the formation of salicylate was differed in the degradation of the both compounds. In the degradation pathway of 2NB, salicylate was directly formed from the 2NB by a monooxygenase activity, whereas in the degradation pathway of 4-chloro-2-nitrobenzoate, salicylate was formed due to reductive dehydroxylation of 2,4-dihydroxybenzoate (Prakash et al., 2010).

*Arthrobacter* sp. SPG has also potential to degrade 2NB in soil. Strain SPG efficiently degrades 2NB in sterile as well as non-sterile soil. This strain may be used for bioremediation of 2NB-contaminated sites.

## Conclusion

*Arthrobacter* sp. SPG utilized 2NB as its sole source of carbon and energy and degraded it in broth culture as well as from soil (sterile and non-sterile) under laboratory conditions. The present study clearly indicates that strain SPG degraded 2NB via salicylate and catechol with potential to be used in bioremediation of 2NB-contaminated sites.

## Conflict of Interest Statement

The authors declare that the research was conducted in the absence of any commercial or financial relationships that could be construed as a potential conflict of interest.
